# Magnesium Ion: A New Switch in Tumor Treatment

**DOI:** 10.3390/biomedicines12081717

**Published:** 2024-08-01

**Authors:** Leyi Huang, Renxi Lin, Jiaxi Chen, Yuanlin Qi, Ling Lin

**Affiliations:** 1 Laboratory of Gynecologic Oncology, Fujian Maternity and Child Health Hospital, College of Clinical Medicine for Obstetrics & Gynecology and Pediatrics, Fujian Medical University, Fuzhou 350001, China; lyhuang23@163.com; 2 Department of Biochemistry and Molecular Biology, Fujian Medical University, Fuzhou 350122, China; linrenxi89@fjmu.edu.cn (R.L.);; 3 Experimental Teaching Center of Basic Medicine, Fujian Medical University, Fuzhou 350122, China; 4 Key Laboratory of Brain Aging and Neurodegenerative Disease, Fujian Medical University, Fuzhou 350122, China

**Keywords:** magnesium ion, mitochondria, mitochondrial function, energy metabolism, cancer therapy

## Abstract

The magnesium ion is an essential cation in the human body and participates in numerous physiological activities. A deficiency in magnesium ions is closely associated with tumor development, and supplementation with magnesium ions has been shown to partially inhibit tumor growth. However, the specific mechanisms by which magnesium ions suppress tumor proliferation remain unclear. Currently, studies have revealed that mitochondria may serve as a crucial intermediate link in the regulation of tumors by magnesium ions. Mitochondria might intervene in the proliferation and invasion of tumor cells by modulating energy metabolism and oxidative stress levels. Regrettably, there has been no comprehensive review of the role of magnesium in cancer therapy to date. Therefore, this article provides a comprehensive scrutiny of the relationship between magnesium ions and tumors, aiming to offer insights for clinical tumor treatment strategies involving magnesium ion intervention.

## 1. Introduction

Magnesium ions (Mg^2+^), the fourth most abundant element in the human body and the second most prevalent cation in cellular environments after potassium ions, actively participate in vital physiological processes. They regulate enzymatic reactions involved in substance synthesis, influencing cell growth, differentiation, energy metabolism, excitability transmission in neurons, and cardiac excitability [1]. This is facilitated by magnesium ion transport proteins, with normal plasma magnesium concentrations ranging between 0.75 and 0.85 mmol/L [2]. Factors such as dietary intake, renal excretion, and skeletal muscle storage can impact magnesium homeostasis [3]. As a calcium ion antagonist, magnesium ion deficiency in neurons leads to increased activity of *n*-methyl-*d*-aspartic acid receptor (NMDA), causing excessive influx of calcium ions, reactive oxygen species (ROS) production, and mitochondrial damage. This heightened neuronal excitability is implicated in various diseases [4], including Parkinson’s and Alzheimer’s diseases, migraine, anxiety, and depression [3].

Due to the critical role of magnesium in physiological functions, some researchers have begun to consider the correlation between magnesium and cancer. Interestingly, besides magnesium ions, certain metal ions are also believed to be closely associated with cancer therapy. A recent study from China found that iron promotes DNA damage repair, thereby promoting platinum resistance in ovarian cancer; thus, iron depletion may emerge as a new direction for ovarian cancer treatment [5]. Similarly, copper can induce oxidative stress, inhibit the ubiquitin–proteasome system, and promote tumor cell death through mechanisms like “cuproptosis” [6]. Furthermore, copper can serve as a nanocarrier to deliver therapeutic drugs, exerting anti-tumor efficacy. Therefore, it is reasonable to speculate that magnesium may also hold significant potential in cancer therapy.

In 1969, Bois et al. [7] successfully induced thymic lymphoma in rats through a magnesium-deficient diet. In 1989, Kurata et al. [8] confirmed in mice that magnesium supplementation in the diet effectively reduced the incidence of liver cancer, while a magnesium-deficient diet could induce tumor formation. A higher magnesium intake in the diet (400 mg/day) has been shown to reduce the incidence of colorectal cancer in postmenopausal women, and elevated blood magnesium levels are negatively correlated with the risk of colorectal cancer in females [9]. Moreover, recent research by Lötscher et al. [10], published in *Cell*, indicated that mice fed a magnesium-deficient diet exhibited significantly reduced efficacy of chimeric antigen receptor T-cell immunotherapy (CAR-T) against tumors, and B-cell lymphoma patients with lower serum magnesium levels had a shorter survival period.

As a cofactor for DNA repair enzymes, magnesium plays a crucial role in maintaining genomic stability and regulating cell proliferation, differentiation, and apoptosis. Magnesium deficiency may compromise DNA stability, contributing to tumor initiation and poor prognosis [11,12]. Yan’s team reported that magnesium glycinate inhibits liver cancer cell proliferation by blocking the mitogen-activated protein kinase (MAPK) signaling pathway in vitro [13]. Additionally, magnesium supplementation can inhibit the transcription of genes related to liver cancer cell proliferation by dephosphorylating p-Smad2/3 and blocking the transforming growth factor β (TGF-β) signaling pathway [14].

Meanwhile, magnesium ions play a crucial regulatory role in mitochondrial function. According to the research conducted by Koning et al. [15], magnesium sulfate can safeguard the mitochondrial respiratory chain, concurrently reducing the generation of ROS and inflammatory factors. Additionally, magnesium glycyrrhizinate is effective in preserving mitochondrial membrane potential and ultrastructure, while mitigating mitochondrial DNA (mtDNA) damage [16]. Given the pivotal role of mitochondrial oxidative stress and mtDNA damage in tumor progression [17], magnesium ions hold promise as a novel target for cancer therapy. Further investigation into their potential mechanisms in anticancer treatment is anticipated to offer new therapeutic avenues and hope for cancer patients.

## 2. Methods

To identify eligible studies for this review, we performed a computerized search of the PubMed and Web of Science databases, covering all publications available up to December 2023. Our search employed keywords including “magnesium”, “cancer”, “mitochondrial”, and their synonyms. Moreover, we scrutinized the reference lists of pertinent articles and reviews to identify studies not indexed in these databases. We meticulously evaluated all abstracts and full-text articles, selecting those deemed relevant for screening and inclusion in this review.

## 3. Magnesium Ion Transport Proteins and Their Implications in Tumor Development

Mg^2+^ is predominantly distributed within cellular compartments, and its intracellular distribution and regulation rely heavily on magnesium ion transport proteins located on cellular and organelle membranes. Currently, major magnesium ion transport proteins include members of the transient receptor potential melastatin (TRPM) channel protein family, the human solute carrier (SLC) superfamily, magnesium transporter (MagT) proteins, cyclin M (CNNM) family proteins, and mitochondrial RNA splicing 2 (Mrs2) family genes.

### 3.1. TRPM Family

The TRPM family is widely expressed in numerous mammalian cells and is implicated in various biological functions, including redox reactions, inflammation, and insulin secretion [18]. This channel protein family comprises numerous members with varying permeabilities to calcium ions (Ca^2+^) and magnesium ions (Mg^2+^). TRPM7 and TRPM9 play crucial roles in intestinal magnesium ion absorption [19]. Moreover, the TRPM family participates in tumorigenesis by regulating the calcium ion balance [20], modulating cellular oxidative phosphorylation levels [21], and influencing ROS generation [22] within mitochondria.

In prostate cancer, the expression of TRPM8 and TRPM2 is significantly greater in tumor cells than in normal cells, whereas in lung cancer, TRPM5 upregulation is predominant [23]. Additionally, the TRPM family plays a significant role in various cancers, such as breast cancer, pancreatic cancer, and melanoma, and it is a potential target for antitumor therapies [18,23]. The TRPM family may provide insights into its potential as a therapeutic target for combating tumorigenesis.

### 3.2. SLC Family

The SLC family comprises crucial membrane transport proteins on the cell membrane and consists of 52 subfamilies with more than 400 members. These proteins are widely distributed on cell membranes and various organelle membranes and function as typical transmembrane proteins [24]. The SLC41 protein family facilitates the transmembrane transport of various metal ions, glucose, lipids, neurotransmitters, and drugs [25], thus playing a crucial role in the development of various diseases, including metabolic disorders, neurological diseases, and tumors [26].

The SLC41 family was discovered in 2003 and comprises SLC41A1, SLC41A2, and SLC41A3 [26]. The SLC41 family is capable of transmembrane transport of Mg^2+^ and, along with other magnesium ion transporters, such as TRPM7, participates in the regulation of Mg^2+^ homeostasis within the organism [27]. Variations in the SLC41A1 gene within the PARK16 locus in Parkinson’s disease patients have a significant impact on the pathogenesis of Parkinson’s disease as well as on ion homeostasis and the function of dopamine neurons [24,26]. Additionally, the overexpression or downregulation of SLC41A1 is closely associated with the occurrence of preeclampsia and renal diseases [24]. A study by Njiaju et al. [28] reported the association between SLC41A2 and chemotherapeutic drug sensitivity, in which knocking down SLC41A2 significantly increased the sensitivity of tumor cells to paclitaxel. Conversely, high SLC41A3 expression is closely correlated with poor prognosis in hepatocellular carcinoma patients, leading to a significantly shorter survival period in patients with elevated SLC41A3 expression than in those with normal SLC41A3 expression [29]. In summary, the SLC41 family participates in the onset of various diseases through the regulation of Mg^2+^ homeostasis and has emerged as a potential target for drug therapy.

### 3.3. MagT Family

The MagT protein family primarily includes MagT1, which is widely expressed in various human organs and tissues. It exhibits high similarity with human tumor suppressor candidate 3 (TUSC3). Both MagT1 and TUSC3 function in magnesium ion transport and protein N-terminal glycosylation. MagT1 was identified as a crucial target gene for X-linked immunodeficiency with magnesium defect (XMEN) disease [30]. The absence of the MagT1 protein leads to impaired *n*-glycosylation of platelet glycoproteins, resulting in platelet dysfunction and accelerated arterial thrombosis formation [31]. Simultaneously, the MagT1 protein regulates the intracellular magnesium ion concentration, influencing macrophage polarization and immune cell function [32].

MagT1 also plays a significant role in regulating the growth of tumor cells. Zheng et al. [33] reported a close correlation between MagT1 expression and poor prognosis in patients with colorectal cancer, with patients expressing lower levels of MagT1 experiencing longer survival. In breast cancer research, Li et al. [34] discovered that downregulation of MagT1 protein expression significantly inhibited the progression of breast cancer by suppressing Ki67. Additionally, MagT1 has been identified as an essential gene for cervical cancer cell proliferation [35], and low MagT1 expression inhibits the growth of cervical cancer cells and promotes cell apoptosis [36].

### 3.4. CNNM Family

The CNNM protein family is primarily distributed in brain tissues, kidneys, and intestines. As cyclin M is a conserved Mg^2+^ transport protein, the main function of this protein family is to transport magnesium ions out of cells. Additionally, the CNNM family proteins promote the absorption of Mg^2+^ in the intestines and kidneys [37]. The mechanism of magnesium transport by the CNNM family is complex. According to Bai et al. [38], the CNNM family proteins were regulated by the phosphatase of regenerating liver (PRLS), which is closely associated with tumor metastasis. CNNM selectively binds to the TRPM7 protein, thereby facilitating Mg^2+^ uptake. The intricate role of the CNNM family in Mg^2+^ transport is crucial for maintaining the intracellular Mg^2+^ balance [37]. Dysfunction of the CNNM family proteins often leads to hereditary hypomagnesemia, resulting in conditions such as hypertension, pulmonary arterial hypertension, epilepsy, Parkinson’s disease, and other disorders [39]. In colon cancer tissues, the expression of the CNNM4 protein is significantly decreased compared to that in normal tissues. Critically, a lower level of CNNM4 implies a greater degree of malignancy of the tumor [40].

### 3.5. MRS Family

The Mrs2 protein is a magnesium ion transport protein located on the mitochondrial membrane. When stimulated by the glycolysis end-product lactate, Mrs2 transports magnesium ions stored in the endoplasmic reticulum to the mitochondria [41]. The spatiotemporal changes in magnesium ions within cells induced by Mrs2 result in reduced mitochondrial oxygen consumption and increased ROS production [42]. Using electron microscopy, He et al. [43] discovered that in addition to Mg^2+^, Mrs2 also serves as a nonselective channel for calcium ions and can permeate sodium and potassium ions. The well-known Warburg effect demonstrates that most tumor cells exist in a microenvironment characterized by lactate accumulation. Therefore, subcellular magnesium ion transport mediated by Mrs2 is likely to become a crucial “switch” in regulating tumor cell growth [44].

Furthermore, Mrs2 plays a significant role in regulating mitochondrial function and cellular metabolism. According to the latest research by Madaris et al. [45], in mice induced with a classic Western diet (a high-sugar, high-fat, high-cholesterol diet), knocking out the mitochondrial Mrs2 channel effectively inhibited Western diet-induced obesity, metabolic syndrome, and spontaneous tumor incidence. This difference may be due to the loss of Mrs2, leading to the reprogramming of systemic energy metabolism regulated by hypoxia-inducible factor-1α (HIF-1α) in the liver and adipose tissue, enhancing mitochondrial activity, reducing hepatic fat accumulation, and inducing browning of white adipose tissue.

**Figure 1:**
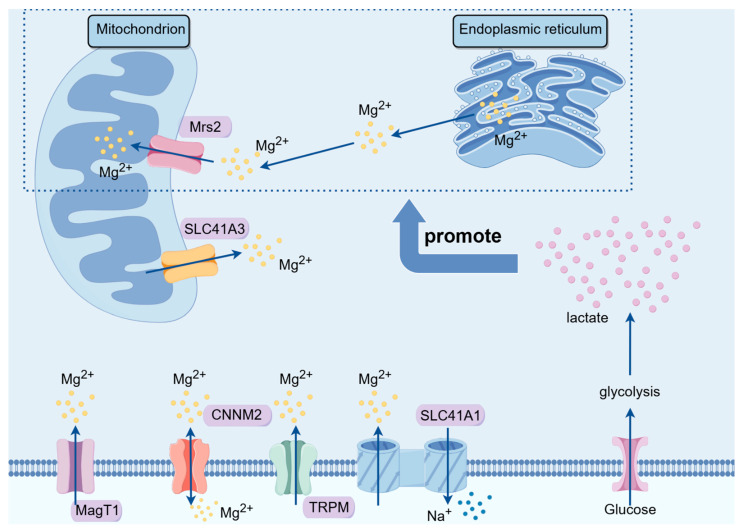
Magnesium ion transporters regulate intracellular magnesium ion levels. On the cell membrane, MagT1 and TRPM7 primarily mediate the transport of magnesium ions into the cell, while CNNM2 is a bidirectional magnesium ion transporter. SLC41A1 functions as a sodium/magnesium exchanger. Meanwhile, on the mitochondrial membrane, there are two magnesium ion transporters. Mrs2 is mainly responsible for transporting magnesium ions from the endoplasmic reticulum into the mitochondria in response to lactate stimulation, whereas SLC41A3 is responsible for transporting magnesium ions out of the mitochondria. **Abbreviations:** TRPM, transient receptor potential melastatin channel protein family; SLC, human solute carrier superfamily; MagT, magnesium transporter proteins; CNNM, cyclin M family proteins; Mrs2, mitochondrial RNA splicing 2 family genes. By Figdraw 2.0.

Due to the unique structural location of Mrs2 and its crucial role in subcellular magnesium ion transport and mitochondrial metabolic functions, Mrs2 has a great potential for elucidating the mechanism of action of magnesium ions against cancer. Mrs2 may serve as a new target for energy metabolism-related tumor therapy (Figure 1).

## 4. The Impact of Magnesium Ions on Tumor Development

### 4.1. Breast Cancer

Breast cancer is a significant gynecological tumor. Interestingly, high magnesium intake improves the prognosis of breast cancer. A study involving 1170 breast cancer patients from New York State found that dietary magnesium intake is negatively correlated with overall mortality [46]. This correlation may be due to the protective effects of magnesium ions on the cardiovascular system, as well as their roles in maintaining genomic stability [47], regulating cell differentiation, proliferation, and apoptosis, as well as preventing angiogenesis [48].

The inhibitory effect of magnesium on breast cancer has also been confirmed in basic experiments. A study by Yang et al. [49] found that increasing the intracellular Mg^2+^ concentration in breast cancer cells, induced by methyl jasmonate, led to a decrease in the expression of the magnesium ion transporter TRPM7, an increase in ROS levels, and the promotion of apoptosis in breast cancer cells. In animal models, knocking out the magnesium ion transporter MagT1 significantly downregulated Ki67, thereby markedly inhibiting the progression of breast cancer [34]. Additionally, a clinical controlled study found that plasma magnesium levels in female breast cancer patients were significantly lower than those in the normal population [50]. Therefore, we have reason to believe that magnesium ions play an important role in the occurrence and development of breast cancer, and that appropriate dietary supplementation of magnesium could effectively inhibit the progression of breast cancer.

### 4.2. Colorectal Cancer

In colorectal cancer research, many investigators have found that magnesium intake greatly influences the risk and prognosis of the disease. According to Gorczyca et al. [51], daily supplementation of 400 mg of magnesium can reduce the incidence of colorectal cancer in postmenopausal women. Additionally, Polter et al. [52] identified through a prospective study that serum magnesium levels were negatively correlated with colon cancer risk among female participants. Moreover, higher magnesium intake effectively improves chemotherapy-induced peripheral neuropathy in colorectal cancer patients [53], Furthermore, Wesselink et al. [54] discovered through a large multicenter prospective cohort study that magnesium intake lowers overall mortality in colorectal cancer patients. This effect may be attributed to magnesium’s crucial role in regulating the synthesis metabolism of 25-hydroxyvitamin D [55]. High levels of magnesium and 25-hydroxyvitamin D inhibit inflammation and enhance patient survival rates [56,57].

In basic research, Li et al. [58] proposed that magnesium supplementation can induce apoptosis in colorectal cancer cells by activating the caspase-3 pathway. This finding was supported by animal experiments, where continuous magnesium injection for three weeks inhibited subcutaneous tumor growth in nude mice, and hematoxylin–eosin staining (HE) confirmed significant apoptotic features in the tumors of magnesium-supplemented mice [58]. These results provide a theoretical basis for the clinical use of magnesium-related drugs in the treatment of colorectal cancer, demonstrating the potential clinical value of magnesium ion therapies.

### 4.3. Other Cancers

In addition to breast cancer and colorectal cancer, magnesium ions have also been shown to be associated with many other common cancers. A prospective cohort study by the Shrubsole team from the United States found that higher dietary magnesium intake is associated with a lower risk of liver cancer [59]. Additionally, an increase in magnesium concentration in lung cancer tissues can enhance the anti-tumor immunity of T cells [10]. In cell experiments, magnesium can effectively inhibit ovarian cancer and osteosarcoma cells [60,61]. However, these basic research findings still require further clinical trials to confirm their therapeutic efficacy.

## 5. Mechanisms of Magnesium Ion Therapy in Tumor Treatment

### 5.1. The Impact of Magnesium Ions on Mitochondrial Function

The intracellular magnesium ion distribution is regulated primarily by various magnesium ion transporter families. Mrs2 and solute carrier family 41 member A3 (SLC41A3) are the main magnesium transporters on the mitochondrial membrane. In 2016, Mastrototaro et al. [62] first observed that the overexpression of SLC41A3 in human embryonic kidney 293 (HEK293) cells led to a 60% increase in mitochondrial magnesium ion efflux compared to that in normal cells. SLC41A3 functions as a Na^+^/Mg^2+^ exchanger, and the efflux of magnesium ions depends not only on the sodium ion concentration but also on temperature. At a temperature of 16 °C, the ion flow nearly disappears [62]. Moreover, overexpression of SLC41A3 may deplete mitochondrial magnesium ions, thereby impairing cellular respiration and reducing mitochondrial ATP production [63]. The endoplasmic reticulum and mitochondria are important storage sites for magnesium ions within cells, and another mitochondrial magnesium ion transporter, Mrs2, acts as a messenger facilitating magnesium ion transfer between these two organelles. Under lactate stimulation, Mrs2 can transport magnesium ions from the endoplasmic reticulum to the mitochondria, further stimulating mitochondrial ROS production [41].

The mitochondrial magnesium ion regulation system composed of SLC41A3 and Mrs2 allows cells to flexibly adjust magnesium ion levels within mitochondria under different physiological and environmental conditions to meet the metabolic needs of the cell. The functions and regulatory mechanisms of Mrs2 and SLC41A3 provide essential safeguards for maintaining cellular energy balance and normal metabolism, underscoring their irreplaceable roles in cell survival and functional maintenance.

#### 5.1.1. The Impact of Magnesium Ions on Mitochondrial Energy Metabolism

In 2016, Ha et al. discovered [64] that magnesium-rich seawater effectively increases the activity of mitochondrial enzymes and the expression of mtDNA in adipocytes, suggesting a potential regulatory and antiobesity role of magnesium ions. As a crucial co-factor for many key enzymes in glucose metabolism pathways, magnesium ions play a significant regulatory role in the energy metabolism of mitochondria. Free magnesium ions can indirectly stimulate the dephosphorylation of pyruvate dehydrogenase (PDH) by pyruvate dehydrogenase phosphatase, thereby upregulating the activity of the pyruvate decarboxylase component within the pyruvate dehydrogenase complex (PDHC). This, in turn, affects ATP synthesis by enhancing the activity of the rate-limiting enzyme in the tricarboxylic acid cycle, the α-ketoglutarate dehydrogenase complex (OGDC), ultimately regulating cellular energy metabolism [65].

#### 5.1.2. The Impact of Magnesium Ions on Oxidative Stress

In both hypertonic and hypotonic environments, magnesium ions and potassium ions play a protective role in the permeability of the mitochondrial outer membrane, effectively safeguarding mitochondrial activity [66]. Conversely, magnesium deficiency inhibits the magnesium transporter protein on the mitochondrial membrane (Mrs2) [67]. As a result, the magnesium ion content within the mitochondria decreases, leading to reduced electron transport chain activity, increased mitochondrial ROS, inhibition of key antioxidant enzymes, and subsequent initiation of oxidative stress [19]. Simultaneously, magnesium ion deficiency, which antagonize calcium ions, increases intracellular calcium overload, activating numerous calcium-dependent kinases and proteins, such as nitric oxide synthase and calcium-dependent calcium-binding proteins, further augmenting ROS production [68]. Additionally, magnesium-deficiency-induced ROS overload activates transcription factors such as nuclear factor kappa-B (NF-κB), which induces lipid peroxidation and stimulates the secretion of proinflammatory cytokines, including interleukin (IL)-1, IL-6, and tumor necrosis factor (TNF)-α, triggering inflammatory responses and exacerbating ROS production [69]. Supplementation with magnesium sulfate has been shown to effectively protect newborn neurons by preserving mitochondrial respiration and reducing ROS production and inflammation [15].

#### 5.1.3. Relationships between Mitochondrial Function and Cancer

The well-known Warburg effect suggests that tumor cells preferentially utilize glycolysis for glucose metabolism, indicating a potential association between mitochondrial dysfunction and the pathogenesis of cancer. Due to the limited energy provided by glycolysis, tumor cells require increased mitochondrial activity to meet their energy demands [70]. Hasumi [71] and Lang [72] confirmed a substantial increase in mitochondrial quantity in malignant pheochromocytoma, accompanied by a significant increase in respiratory frequency compared to that in normal cells. Cannino et al. [73] observed an increased number and enlarged volume of mitochondria in tumor cells, with notable structural differences from those of normal mitochondria, including swelling, shrinkage, and alterations in the outer membrane. These abnormalities further impact mitochondrial respiratory function and stimulate the accumulation of lactate.

**Figure 2:**
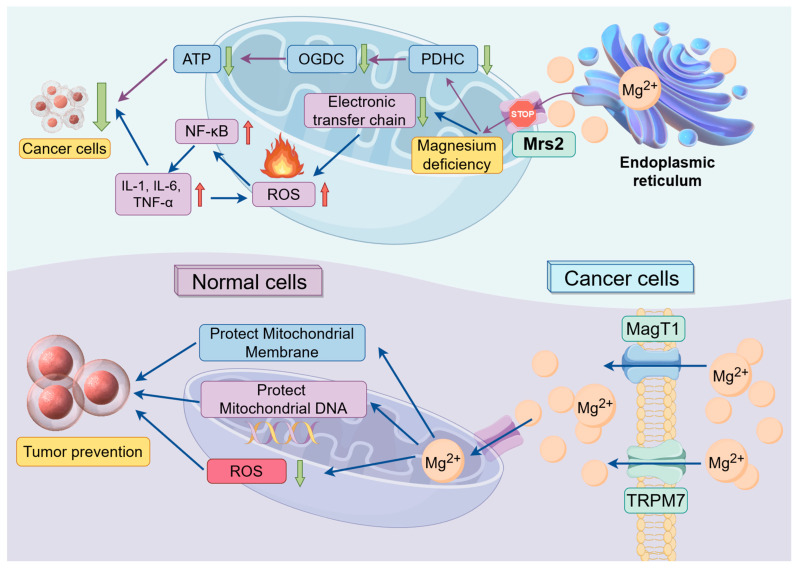
The potential application of magnesium ions in cancer therapy. This figure illustrates the potential application of magnesium ions in cancer therapy. By blocking the Mrs2 protein in tumor cells, the magnesium content in mitochondria will decrease. This deficiency will further reduce electron transport chain activity, increase ROS production, and activate inflammatory responses. For tumor cells, excessive oxidative stress and inflammation will induce cell death. Additionally, mitochondrial magnesium deficiency will lead to reduced ATP production, causing energy deprivation and subsequent cell death in tumor cells. Conversely, supplementing with adequate magnesium can increase intracellular magnesium ion content, enhance mitochondrial magnesium levels, reduce ROS production, and protect mitochondrial membranes and mitochondrial DNA. This helps maintain mitochondrial function stability and prevent tumorigenesis. Green arrows indicate a decrease in the substance content, while red arrows indicate an increase in the substance content. **Abbreviations:** TRPM, transient receptor potential melastatin channel protein family; MagT, magnesium transporter proteins; Mrs2, mitochondrial RNA splicing 2 family genes; PDHC, pyruvate dehydrogenase complex; OGDC, oxoglutarate dehydrogenase complex; ROS, reactive oxygen species; NF-κB, nuclear factor kappa-B; IL, interleukin; TNF-α, tumor necrosis factor. By Figdraw 2.0.

Therefore, mitochondria have emerged as novel targets for cancer therapy. Magnesium can inhibit cancer cell energy supply, promote apoptosis, and impede tumor proliferation and invasion [74] by suppressing mitochondrial respiratory chain activity [75], inducing excessive ROS production [76], damaging mitochondrial DNA [77], and interfering with tumor cell metabolic pathways [78]. Additionally, magnesium supplementation can protect the mitochondrial membrane integrity of normal cells and stabilize mitochondrial DNA, reducing the production of ROS, thereby preventing oxidative stress and inflammatory responses, as well as ultimately contributing to tumor prevention (Figure 2).

### 5.2. Magnesium Ions and Inflammation

Magnesium ions participate in immune responses through various mechanisms. Magnesium maintains the normal function of cell surface proteins on CD8+ T cells and regulates T-cell-mediated immune responses, which are crucial for cancer immunotherapy [10]. Additionally, magnesium is an essential cofactor for the synthesis of immunoglobulins, C3 convertase, and the adhesion of immune cells [79]. Magnesium deficiency can increase chronic inflammation; enhance immune stress; and lead to the activation of TNF, IL-1, and IL-6, thereby stimulating tumor proliferation [68]. Therefore, maintaining adequate magnesium levels is crucial for cancer patients.

### 5.3. Magnesium Regulates Apoptosis in Tumor Cells

According to research by Wei et al. [80], magnesium can activate the adenosine 5‘-monophosphate (AMP)-activated protein kinase (AMPK)/mammalian target of rapamycin (mTOR)/ULK1 pathway, thereby participating in the activation of the autophagy pathway and inducing apoptosis in osteosarcoma cells. Furthermore, since magnesium can degrade into magnesium ions, hydroxide ions, and hydrogen gas, it can partially block the cell cycle, inhibit the proliferation of gallbladder cancer cells, and induce apoptosis [81]. The research of Yuan et al. [82] also confirmed that magnesium isoglycyrrhizinate can inhibit the apoptosis of bladder cancer cells HTB9 and BIU87 and suppress bladder cancer progression by upregulating miR-26b expression and inactivating the NADPH oxidase 4 (NOX4)/NF-κB/HIF-1α signaling pathway. In breast cancer cells, an increase in intracellular magnesium similarly induces apoptosis in MCF-7 cells [49]. Therefore, magnesium may exert antitumor effects by inducing apoptosis.

## 6. Prospects of Magnesium Ions in Tumor Therapy

### 6.1. Clinical Applications of Magnesium in Cancer Therapy

Currently, magnesium ions have been employed as anti-tumor implant materials for interventional therapy. During degradation, magnesium generates magnesium hydroxide, hydrogen gas, and magnesium ions. Magnesium hydroxide alkalinizes the acidic tumor microenvironment, while hydrogen gas and magnesium ions exhibit significant anti-inflammatory and antioxidant effects [83].

An increase in the blood magnesium concentration can inhibit mitochondrial oxidative stress, restraining tumor cell proliferation and invasion [84]. Additionally, magnesium ions can modulate the function of T cells, promoting the activation of immune responses and inhibiting tumor growth [10]. Regrettably, the clinical application of magnesium in cancer therapy remains rather limited, despite some clinical trials having confirmed its inhibitory effects on tumor progression. As a key “switch” in regulating tumor growth, magnesium ions are poised to become a new target for cancer therapy and hold potential for widespread clinical applications in the future.

### 6.2. Other Roles of Magnesium Ions in Cancer Therapy

In addition to directly inhibiting tumor growth, magnesium can be used in the treatment of cancer patients for pain relief, antispasmodic effects, and thrombosis prevention. According to the study by Na et al. [85], intraoperative intravenous injection of magnesium sulfate can effectively reduce the hypercoagulable state in patients undergoing laparoscopic colorectal cancer surgery. Additionally, Wu et al. [86] found that oral administration of magnesium-l-threonate can enhance the analgesic effect of opioids, reduce the required dosage of opioid drugs, and alleviate opioid-induced constipation, thereby improving the quality of life for patients with advanced cancer. For patients undergoing mastectomy, the administration of 250 mL of magnesium sulfate at 50 mg/kg prior to anesthesia induction can reduce postoperative analgesic requirements and alleviate postoperative pain to some extent [87]. Furthermore, because magnesium ions relieve smooth muscle spasms, postoperative administration of magnesium sulfate can help alleviate catheter-related bladder discomfort in surgical patients [88,89].

## 7. Conclusions

**Figure 3:**
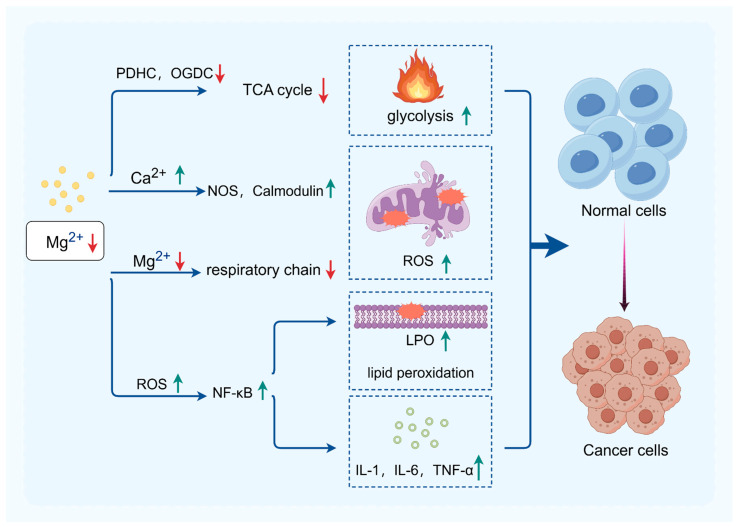
The relationship between low-magnesium ions and cancers. Magnesium deficiency will affect the tricarboxylic acid (TCA) cycle, promoting lactate accumulation. It also leads to elevated calcium ions, which enhances oxidative stress. The production of ROS will further upregulate NF-κB, stimulating the production of inflammatory factors and lipid peroxidation. These effects may potentially lead to tumorigenesis. Green arrows indicate a increase in the substance content, while red arrows indicate an decrease in the substance content.

In general, the dysfunction of mitochondria is closely associated with the occurrence and progression of tumors. Given the pivotal role of mitochondria as the “engine” in cellular energy metabolism, targeted regulation of mitochondrial function is poised to emerge as a novel direction in cancer therapy. Magnesium ions can play a role in the prevention and treatment of tumors by influencing ROS production, oxidative stress levels, mtDNA stability, and the activity of key mitochondrial metabolic enzymes. Magnesium ion therapy holds promise as a novel therapeutic modality for metabolic tumors, offering advantages such as low toxicity and anti-inflammatory properties, thus exhibiting significant clinical application potential (Figure 3).

## Data Availability

Not applicable.

## Abbreviations

ROS	Reactive oxygen species
TRPM	Transient receptor potential melastatin channel protein family
SLC	Human solute carrier superfamily
MagT	Magnesium transporter proteins
CNNM	Cyclin M family proteins
Mrs2	Mitochondrial RNA splicing 2 family genes
PDHC	Pyruvate dehydrogenase complex
OGDC	Oxoglutarate dehydrogenase complex
NOS	Nitric oxide synthase
LPO	Lipid hydroperoxide
NF-κB	Nuclear factor kappa-B
IL	Interleukin
TNF	Tumor necrosis factor
HE	Hematoxylin–eosin staining
